# Impact of CBD and hemp oil use on drug test results: a systematic review

**DOI:** 10.1007/s00414-026-03790-5

**Published:** 2026-04-07

**Authors:** Duygu Yeşim Ovat, Ezgi Bezci, Kemal Balıca, Umut Kırlı, Serap Annette Akgur

**Affiliations:** Ege University Institute on Drug Abuse, Toxicology and Pharmaceutical Science, Bornova, Izmir, Türkiye

**Keywords:** Cannabis, Cannabidiol, CBD oil, Delta-9-THC, Drug testing, Hemp oil

## Abstract

**Background:**

Hemp-derived products, especially CBD (cannabidiol) oil and hemp oil, have shown a notable increase of use in recent years. CBD oil is a compound derived from the cannabis plant, whilst hemp oil is a product derived from hemp seeds. Trans-Δ9-tetrahydrocannabinol (THC) in cannabis is responsible for the psychoactive effect, whereas CBD, having the same molecular weight, is a non-psychoactive compound with analgesic, antiepileptic, antibacterial and anti-inflammatory effects. The rapid uptake of these products has raised concerns about unintended cannabinoid findings (THC, 11-OH-THC and THC-COOH etc.) on drug tests, especially in workplaces, road safety applications, and anti-doping programs. Although many CBD and hemp oils are marketed as “THC-free,” trace amounts of THC may be present in some products due to manufacturing variability, mislabeling, or contamination. These trace amounts may yield detectable levels in biological matrices or may cause cannabinoids findings. Regulatory frameworks for THC in cannabis products exist in several jurisdictions, but oversight of CBD-containing oils remains heterogeneous, creating gaps in quality control and labeling.

**Methods:**

This systematic review aimed to evaluate whether the use of CBD and hemp oil may lead to cannabinoid-positive results in drug testing, potentially causing suspicion of cannabis use. The research design was prepared according to the PRISMA guidelines. The review included studies published in English between March 2014 and March 2024 that met the search criteria based on the keywords “psychoactive effects”, “forensic toxicology”, “THC positive”, “delta-9-tetrahydrocannabinol”, “delta-9-THC”, “test”, and “analysis” paired with the terms “CBD Oil” and “Hemp Oil” separately in the PubMed, WOS, and SCOPUS databases.

**Results:**

The literature search yielded 477 results, 12 of which met the inclusion criteria. Eight of the 12 studies included evaluate of the possibility of positive cannabinoid findings or negative results in biological materials as a result of CBD and hemp oil use in humans. Five of these studies reported that the use of these oils would not create a positive result above the “limit of detection (LOQ) or cut-off levels” in the drug tests for THC. Although concentrations above the limit were not encountered, it is noteworthy, that trace levels or those close to the detection limits of cannabinoid levels were obtained. There are four studies designed to evaluate the conversion of CBD to THC in human metabolism after the use of oil products and two out of four studies emphasize that there will be no biotransformation or extraction-induced transformation after the use of CBD and hemp oils.

**Conclusions:**

This systematic review addresses current evidence on the occurrence and underlying causes of cannabinoid-positive drug test results following the consumption of CBD and hemp oils, highlighting critical gaps in analytical testing strategies and regulatory oversight. Positivity mainly arises from the product content (e.g., undeclared or excessive THC, mislabeling, and contamination), the matrix, the administration route, and the sensitivity and specificity of the analytical methods. The potential for CBD to convert to THC during manufacturing or in the human body may pose legal risks for individuals using CBD or hemp oil in countries with zero-tolerance policies.

## Introduction

The cannabis plant, with its extensive historical roots, can trace its beginnings to the emergence of the earliest agricultural communities in Asia [1]. The cannabis plant has been used as a source of fibers, food, oil, and medicine, as well as for recreational and religious purposes due to its psychoactive effects over the centuries [2, 3]. Today, the cannabis plant is generally called *hemp* when used as fiber and oil and called *marijuana* when used for its psychoactive effects. Cannabis is a plant containing more than 500 different components, of which more than 100 are cannabinoids, the so-called phytocannabinoids (natural cannabinoids) [4]. The most well-known and studied phytocannabinoids in cannabis plants are trans-Δ9-tetrahydrocannabinol (THC) and cannabidiol (CBD). THC is the active component responsible for the psychoactive effect of cannabis, which causes changes in perception, mood, consciousness, and behavior [5]. CBD, which has the same molecular weight as THC but has no proven psychoactive or addictive effects yet. Cannabis use or exposure is typically confirmed by detecting THC and its key metabolites, such as THC-COOH and 11-OH-THC, depending on the biological matrix [6].

The two primary product categories dominating the cannabis market are ***hemp oils*** and ***CBD oils***. The term “hemp oil” is a broad classification encompassing all oils extracted from cannabis, including both seed and plant sources. This general term may or may not include oils containing cannabinoids like CBD. However, products labeled as ‘hemp oil’ often refer to hemp oil with trace amounts of CBD or no CBD at all [7]. However, there is a possibility of THC contamination in the oil due to potential seed contamination with leaf and resin material, which contains the other psychoactive cannabinoids [8]. In contrast, CBD oils, the most common and easily accessible CBD product, are cannabinoid-rich (CBD, CBG (Cannabigerol), CBN (Cannabinol), CBC (Cannabichromene) etc.) products derived from mature cannabis plants. CBD oils are essentially concentrated extracts obtained from cannabis flowers or leaves, which are then dissolved in edible oils such as sunflower, hemp, or olive oil [9]. Today, CBD is commonly used as a food supplement and is also marketed by the Food and Drug Administration (FDA) as a therapeutic agent [10]. CBD-containing products have been reported to be used for coping with pain, stress, anxiety, sleep disorders, epilepsy, neurological disorders, and improving quality of life [11, 12]. Recent market analyses indicate a substantial increase in the popularity and consumption of hemp-derived products worldwide [13–15]. Based on the latest data, in the United States, a national survey reported that approximately 14% of American people have used CBD products, with younger adults and those in urban areas being the most frequent users [16]. The European CBD market was valued at approximately €1.6 billion in 2023 and is projected to reach over €3 billion by 2026, reflecting both consumer demand and expanding legal access [14].

There are different legal regulations around the world depending on the purpose of cultivation and use of the cannabis. The recreational use of marijuana is prohibited in most countries (Malaysia, United Arab Emirates, Indonesia, Japan, Singapore, Saudi Arabia, and Türkiye); however, some countries (Canada, Georgia, Germany, Malta, Mexico, South Africa, Mexico, Thailand, and 24 states in the United States and Australia) have chosen to decriminalize the possession of certain amounts of marijuana [17, 18]. Nowadays the recreational use of marijuana and the use of non-prescription cannabis products (CBD and hemp oils, extracts, hemp seeds, hemp milk, etc.) as a food and health supplements is on the agenda of some countries. The European Commission has received more than 150 applications for CBD to be treated as a novel food, 19 of which are currently on hold for safety assessment by the European Food Safety Authority (EFSA) due to data gaps and uncertainties about potential hazards associated with CBD [19]. One of these gaps is that the CBD and THC content of these products may not be indicated on the labels or may contain different amounts or different ingredients. The FDA has issued warning letters to CBD sellers at different times regarding the ingredients of hemp-containing food products sold on the market, warning sellers that CBD should not be legally “marketed” as a food or dietary supplement because it can be a Schedule-I substance under the Controlled Substance Act [20].

Different regulations have been implemented for the THC content of hemp products, especially CBD oils. Regulatory limits for THC vary, in the USA, the sale of all cannabis products containing up to **0.3% THC** has been authorized since 2018 [21]. Although the Council of Europe authorized the sale of all cannabis products containing up to 0.3% THC in 2021, the actual control is left to the countries’ own legal regulations; in Switzerland, Italy, and Australia, it is legal to contain up to 1% Δ9-THC in these products [22]. However, there are no uniform regulations concerning this subject in EU countries or in the world [23].

Beyond regulatory discrepancies between countries, recent studies have raised critical concerns regarding the actual cannabinoid content of commercially available CBD products, particularly the unintentional presence of THC in the event of involvement in a road traffic accident, or in workplace drug testing (WDT) in many countries [24–28]. A positive drug test result is more likely when the hemp-derived product does not meet the legal THC limit of less than 0.3% as defined by the 2018 Farm Bill. For this reason, several U.S. military branches, government agencies, and private employers have issued warnings or imposed restrictions on the use of CBD products among their personal [29, 30]. In a study by Lindekamp et al., THC and structurally related cannabinoids were detected in 21 out of 26 analyzed CBD oil products. Notably, Δ9-THC concentrations in these samples ranged from 5 to 1576 mg/kg, significantly exceeding the legal limits commonly used [31–33]. Comparable findings have been documented by Johnson et al. (2022), and Pavlovic et al. (2018), further emphasizing the widespread variability in product content [34, 35]. In a large-scale assessment by Johnson et al., 80 CBD oils were tested, revealing that 64% (51 products) contained THC at concentrations ranging from 0.008 mg/mL to 2.071 mg/mL, with a mean of 0.620 mg/mL and a median of 0.640 mg/mL. Significantly, even among products labeled as “THC Free,” 24% (5 out of 21) were found to contain THC between 0.015 and 0.656 mg/mL. Accidental Δ9-THC exposure may lead to health and safety risks, and can have legal and professional consequences, including issues related to employment, driving, military service, sports eligibility, or child custody [34]. Therefore, within the uncontrolled market landscape of CBD products where unreliable labeling and unverified composition are prevalent the detection of low-concentration THC, even from purportedly ‘THC-free’ products, carries significant implications for forensic drug testing frameworks, particularly in zero-tolerance jurisdictions. Lachenmeier et al. analysed CBD oils on the German market and found significant levels of THC in the products. A notable portion of the tested CBD oils contained THC levels that exceeded the safety limits. Their data showed that 95 out of 219 products contained THC above the safe Acute Reference Dose (ARfD), and 18 products even exceeded the level where adverse effects can begin to appear (Lowest Observed Adverse Effect Level, LOAEL). This finding suggests that consumers are exposed to inherently incompatible products in the marketplace [36]. It is concerning that there is not enough clinical research on the effects of using these oils together with alcohol and other psychoactive drugs and the problems that may be encountered due to their side effects, especially among adolescents, young adults and elderly [9, 37, 38].

In addition to these uncertainties (lack of policy, legislation, regulations, and content analysis of cannabis products), there is also some disagreement regarding the instrumental methods for determining the cannabinoids in these products. In the ‘Cannabis Law Report’ published by the European Union Drug Agency (EUDA) in 2023, the two most abundant cannabinoids in the cannabis plant were highlighted. These are non-psychoactive substances called *“tetrahydrocannabinolic acid (THCA)”* and *“cannabidiolic acid (CBDA)”*, which undergo decarboxylation. It has been stated that when activated via decarboxylation, they convert into THC and CBD. Decarboxylation predominantly occurs upon exposure to heat, such as during smoking, vaporization, or the preparation of edible cannabis products. In particular, the conversion of THCA to THC may lead to psychoactive effects, including euphoria, relaxation, and altered sensory perception [39]. These transformation processes may influence the cannabinoid profile of cannabis-derived products and consequently affect their pharmacological and toxicological outcomes.

Overall, the use of CBD and hemp oils is under scrutiny for judicial and clinical reasons, with respect to some established factors (i) plant genetics (ii) extraction methods used to determine the content of the product in the analysis, (iii) heterogeneous regulatory standards, (iv) false statements in the label information on products offered by manufacturers, and (v) the presence of THC and CBD’s potential transformation during processing, storage, or gastric conditions [12, 40–43]. The aim of this systematic review is to evaluate drug testing outcomes in biological specimens from individuals using CBD and hemp oils, with a particular focus on the detection of cannabinoids such as Δ9-THC, 11-OH-THC, and THC-COOH.

## Materials and methods

### Research strategy and report Identification

The research was conducted using the keywords “psychoactive effects,” “forensic toxicology,” “THC positive,” “delta-9-tetrahydrocannabinol,” “delta-9-THC,” “test” and “analysis” paired with the terms “CBD Oil” and “Hemp Oil” separately on PubMed, WOS and SCOPUS databases in the last decade. During the search by keywords, it was aimed to prevent possible data loss by covering all sections such as title, keywords, abstract, etc. with the “all fields” search option in the databases. The data obtained were stored in xls extension and the data was obtained from the three databases were combined. Considering the names and DOI numbers of the studies included in the study, duplicate publications were manually removed from the excel file. After removing duplicate publications, the remaining publications were included in the evaluation in terms of content relevance. Following duplicate removal, publications were screened for eligibility based on their titles and abstracts. When the information provided in the title and abstract was insufficient to determine eligibility, the full text of the article was reviewed.

### Inclusion and exclusion criteria

#### Inclusion criteria

Reviews, research articles, and case reports published in English between March 2014 and March 2024 that met the search criteria in line with the keywords were included. In addition, studies that were not listed because of filtering while searching with keywords but met the criteria reached through the reference lists of the included studies were also included.

#### Exclusion criteria

We excluded non–peer-reviewed items and formats not suited for evidence synthesis as well as purely analytical method papers without relevance to drug-test interpretation, in-vitro only or animal preclinical studies without translational linkage, and studies on cannabis-derived products for cosmetic/healthcare applications unless their oil formulations were explicitly tested for the potential to trigger THC-positive results in humans. Some of the studies listed after filtering were technically “Commentaries, Letters to the editor, Viewpoints, Erratum, Book chapters” and content-wise “purely analytical, in vitro or animal preclinical studies and reports” were excluded. Studies dealing with materials that were intentionally formulated to contain THC were excluded.

**Fig. 1:**
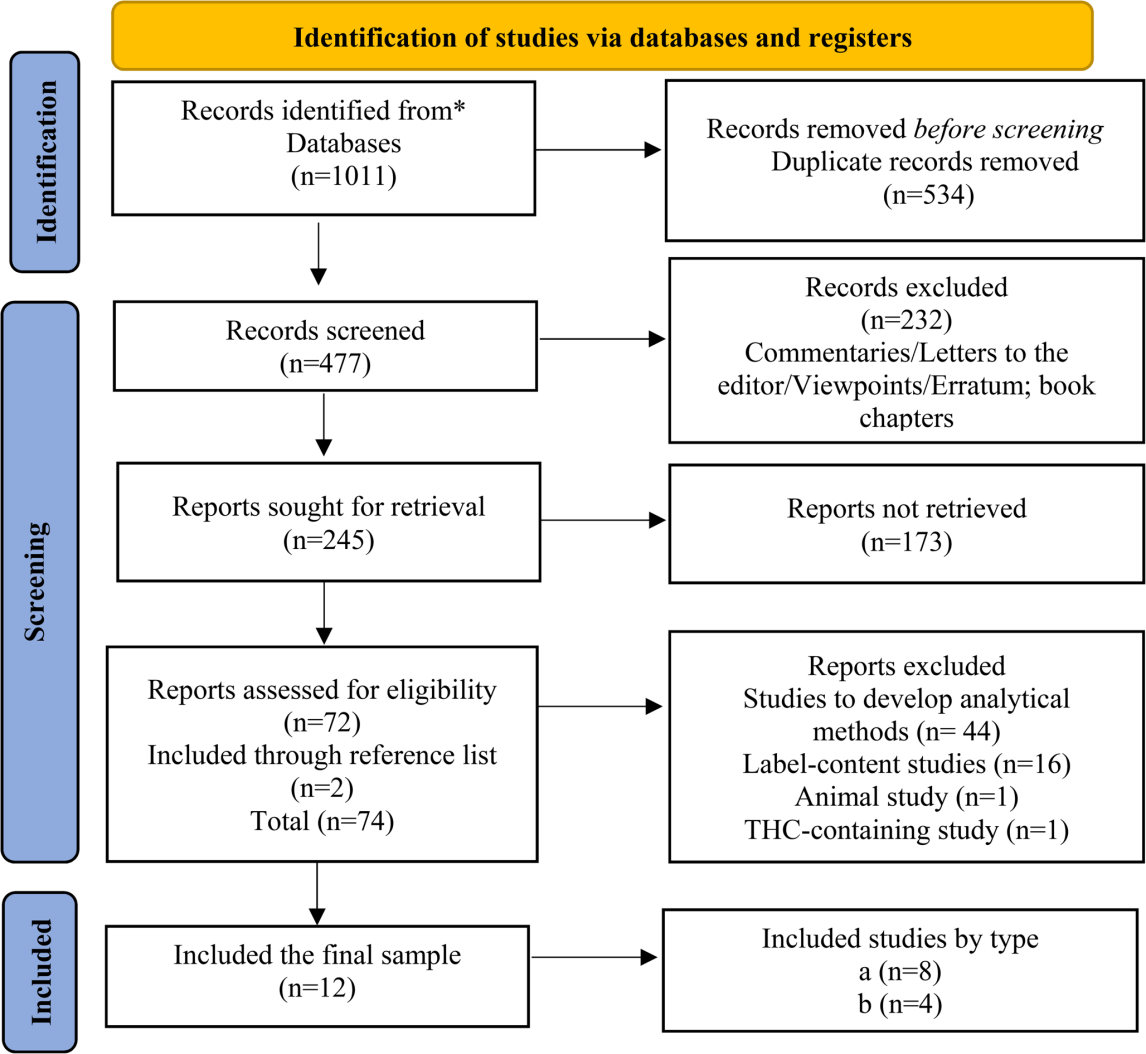
Flow diagram of the literature search (Prisma flow diagram). (**a**): Studies hypothesizing the presence of THC and its metabolites in biological material by oral and dermal ingestion of CBD and hemp oil, (**b**) Studies hypothesizing the conversion of CBD to THC in human metabolism

The research design was prepared according to the PRISMA guidelines. The results of the systematic review are presented as a flow diagram of the literature search (Fig. 1). After the inclusion and exclusion criteria were determined, the data obtained were evaluated separately and then crosschecked. The search protocol used in this study yielded 477 references. The abstracts of the filtered studies were reviewed, and 232 were excluded because of their technical structure and independence of purpose. Noted that 245 studies were not included. The following points (i-iv) were used to identify and exclude studies deemed “inappropriate” :

i) In vivo and in vitro studies in which CBD and hemp oils are used for therapeutic purposes (such as “Sativex” and “oral-mucosal spray” for the treatment of severe chronic pain, fibromyalgia, autism spectrum disorders, cancer, insomnia, etc.) and designed to measure the efficacy of this treatment are excluded in the first round. When the remaining 173 studies were analyzed in detail, 101 were excluded for the following reasons.
ii) Studies that addressed only the ambiguities in the product labels of CBD and hemp oils offered for sale in the market, the amounts of THC contained, and the legal regulations of countries on this issue were excluded from this review as the second group that was not fit for purpose.
iii) Studies excluded that investigated products containing CBD but not directly in the form of oil or not directly produced from these oils (vape, plant extracts, CBD-containing cosmetics, and food products).
iv) Studies showing that CBD found in CBD and hemp oils can be converted to THC under different device and extraction conditions (temperature and pH) using different analytical techniques (related to agriculture and production processes) have been excluded.

Seventy-two studies were initially included according to the PRISMA screening process. During the reference list screening of these studies, two additional articles that met the inclusion criteria but were not identified during the initial database search were retrieved. With the inclusion of these studies, the total number of eligible studies increased to 74. During the final revision stage, one study was excluded because the evaluated product was intentionally formulated to contain THC. Consequently, 12 studies were retained for the final evaluation. These were included in this review and these studies were evaluated in two separate categories as a) and b);

- The THC results obtained in the biological material by oral consumption or dermal exposure of hemp and CBD oil products were first evaluated under subheading 3.1 (Table 1).
- The conversion of CBD to THC in human metabolism as a result of the use of CBD and hemp oil products are given in Table 2 under subheading 3.2.

**Table 1:** Drug test results after CBD and hemp oil intake in biological materials

Study Code	Ref.	Product Type	Administ-ration	Dose or THC content of the oil (%)	Partici- pants	Sample Type	Method	Cut-off / LOQ	Timing range	Result	Key Points	Country (Legal Status)
S1	Hess et al., 2017 [49]	Hemp creams (oil containing)	Topical	Applied every 2–4 h for 3 d. A: 0.0017% (1.7 ng/mg) B: 0102% (102 ng/mg)	3	Blood, Urine	GC–MS	*Plasma (ng/mL)*; LOD_THC_:0.40 LOD_THC−COOH_:1.6 LOD_11−OH−THC_: 0.28 *Urine (ng/mL)*; LOD_THC−COOH_: 1.2	Every 2.–4. h until 15 h after the last application	THC and THC-COOH were not detected	Frequent topical use did not cause positive test results for cannabinoids. Application on irritated skin did not lead to positive results either.	Germany Not stated
S2	Hayley et al., 2018 [26]	Hemp-derivate oil	Oral	Consumed during 3 w. A: 0.001% (10 mg/kg) B: 0.002% (20 mg/kg)	35	OF, Blood, Urine	Drugwipe screening device for OF LC-MS/MS	*OF (ng/mL);* THC screening Cut-off: 20 *Blood and Urine (ng/L);* LOD_THC−COOH_: 0.2	Between 5.–240. min.	THC and THC-COOH were not detected	Trace amounts of THC-COOH was detected in blood and urine in post consumption.	Australia Max. amount of THC of 10 mg/kg permissible for HSO
S3	Hodson et al., 2019 [45]	Hemp oil	Topical	Consumed ~ 2 ml/day for 6 w. Content was not stated.	10	Hair	GC-MS/MS	LOQ_THC_:0.41 pg/mg LOQ_CBD_:3 pg/mg LOQ_THC−COOH_: 0.24 pg/mg	After 6 weeks	THC, THC-OH, THC-COOH were not detected	Cosmetic use may leave trace amounts of THC.	UK Not stated
S4	Baeck et al., 2019 [48]	Hemp seed products (hemp oil containing)	Oral	Consumed ~ 11.1 µg/day Short-term:7d. Long-term: 12w. Content was not stated.	32 + 2 HSO products	Urine	GC-MS	THC screening cut-off: 25 ng/mL LOQ_THC−COOH_: 2.5 ng/mL	After related terms	THC and THC-COOH were not detected	Consuming HSO does not pose any risk in terms of confirmatory tests for cannabinoids and cannabis abusers cannot attribute positive test results from biological samples to the consumption of such HS products	Korea The THC limit for for Hemp seed oil 10 mg/kg or less
S5	Kladar et al., 2021 [44]	Hemp seed oil (HSO)	Oral	Consumed mg/day Min: 0.00011% (1.11 mg/kg) Max: 0.00081% (8.10 mg/kg)	17 HSO products + participants (not stated)	Not declared	GC-MS	LOQ_CBD_:1 mg/kg LOQ_THC_:1 mg/kg	Not stated	THC and THC-COOH were not detected	The HSO were the second most Δ9-THC containing products at this study. Regarding the younger population (< 18 years), the “total” Δ9-THC exposure in significant number of products of any type exceeds the recommended ARfD (0.4 µg/ kg bw).	EU Limits vary
S6	Meznar et al., 2016 [47]	Hemp products (hemp oil containing cookies)	Oral	Consumed piece of cookies (between ¼ piece and 3 piece) Content was not stated.	12	Urine, Serum	Immuno-assay LC-MS/MS	*Urine*: THC screening cut-off : 50 ng/mL *Serum*: LOQ: not stated	Few hours after ingestion	All of the patients resulted THC positive on urinary drug screening test. Low-level THC in serum (*n* = 6)	The growing promotion of hemp-based foods for health benefits may contribute to confusion and may result in unintentional cannabis intoxication, particularly among older adults.	Slovenia The use of extracts from cannabis plant for medicinal purposes is legal, the use of cannabis are prohibited
S7	Chinello et al., 2017 [46]	Hemp oil	Oral	Concumed 1 teaspoon twice a day for ~ 3 weeks	1 (child: 2 years 7 months)	Urine	Immuno-assay GC-MS	THC screening cut-off: 50 ng/mL LOQ: not stated	0.h, 19.h, 8. day	THC-COOH detected (68 ng/mL)	Δ9-THC was detected at 0.06% concentration in oil by GC-MS. This case emphasizes that even “healthy” products marketed in unconventional medicine are possible sources of harmful effects.	Italy Legal limit for THC in HSO is 0.5%
S8	Mareck et al., 2022 [50]	Hemp products (HSO containing)	Oral	Consumed 10–15 g (2 table spoons) A:3.36 × 10^− 7^% (3.36 ng/g) B:1.43 × 10^− 7^% (1.43 ng/g)	23 hemp products (2 hemp oils) 22 volun-teers	Urine	GC-MS/MS	LOD_THC_: 0.18 ng/mL LOD_THCA_: 0.45 ng/mL	Between 0–32. h (0, 8., 16., 32.h)	THC-COOH detected	Hemp oils can cause doping test risks.	Germany For WADA; all cannabinoids banned (except CBD)

* Any legal limits specified for the study at the time it was conducted were added to the “country (legal status) column”

**The “timing range column” indicates the period during the collection of the samples

***If products were also studied alongside the biological material of the participants in the table in the “participant column”, they are indicated as “products.*”*

****The“Dose and THC content of oil(%)”column indicates the THC content of the oils used in the studies, and those that can be converted to a percentage have been converted.Dosage details have also been added if there is

*HSO* Hemp-seed oil, *OF* Oral fluid, *d* day, *w* week

**Table 2:** Biotransformation of CBD to THC after CBD and Hemp oil intake

Study Code	Ref.	Product Type	Administration	Dose or CBD content of the oil (%)	Partici-pants	Sample Type	Method	Cut-off / LOQ	Timing range	Result	Key Points	Country (Legal Status)
S9	Merric et al., 2016 [52]	CBD oil based solution	Not administered (simulated, in vitro)	4% CBD (∼40 mg/mL) Content was not stated.	None (in vitro) experiment)	Simulated gastric fluid (SGF)	LC-MS/MS	Not stated	5–180 min.	Converted.	CBD can convert to Δ9-THC in acidic SGF	USA Not stated
S10	Crippa et al., 2020 [55]	CBD Oil	Oral	300 mg CBD Content was not stated.	120	Plasma	LC-MS/MS	LLOQ_THC_: 1.5 ng/mL	0–216 h	Not converted.	No conversion of CBD to THC in humans	Brazil Not stated
S11	Mullen et al., 2023 [56]	THC-free CBD oil	Not administered (simulated, in vitro)	30 mg CBD/day 120 mg CBD/day Does not contain THC.	None (in vitro) experiment)	Synthetic gastric fluid (SGF)	LC-MS/MS	LOQ_THC_: 2.5 ng/mL	Not specified	Not converted. In SGF, conversion of CBD to Δ9‑THC depends on formulation	Unlikely to cause positive drug tests	USA CBD products labeled THC-free or < 0.3% THC
S12	Franz et al., 2023 [53]	Water-soluble CBD oil	Oral	Consumed 2 ml CBD liquid (~ 400 mg CBD, ~ 0.24 mg THC in 6 mL ethanol) Measurement of the CBD liquid: 0.012% (0.123 THC mg/mL)	9	Blood, Urine	LC-MS/MS	*Serum;* LOQ_THC_: 0.5 ng/mL LOQ_THC−COOH_: 5 ng/mL *Urine;* LOQ_THC−COOH_: 4.8 ng/mL	*Blood;* Between 0–4 h *Urine;* Between 0–48 h	Converted. Serum negative; THC-COOH detected in urine (max 17.9 ng/mL at 6 h)	High CBD use may cause THC metabolite positivity in urine	Germany CBD products containing 0.2% or more THC are considered illegal.

*SGF* Simulated gastric fluid, *LLOQ* Lower limit of quantification

## Results

### THC test results in biological materials

As shown in Table 1, 8 of the studies (coded S1-S8) included analyses on the possibility of cannabinoid positive results in biological materials as a result of CBD and hemp oil use in humans [26, 44–50, 52]. ***Five of these studies (S1-S5) reported that the use of these oils will not create a positive result above the limit of detection or cut-off levels in the drug tests for cannabinoids***. Although concentrations above the limit were not encountered, it is noteworthy that trace levels or close to the detection limits of THC were obtained in these studies ([26, 44–45, 48, 49]). Study S2 evaluated the effect of hemp-derived oils consumed as food products on drug screening tests in 35 adult volunteers in Australia and New Zealand. Oral fluid, urine, and blood samples from the volunteers treated with CBD oil were analyzed. Following the consumption of THC-containing oil at both low (10 mg/kg) and high (20 mg/kg) doses, no traces of cannabinoids were identified in any of the samples across all time points examined. Trace amounts of 11-nor-D9-tetrahydrocannabinol-9-carboxylic acid (THC-COOH) were detected in blood and urine 4 h after the consumption of oil; however, the results were below the LOQ level (0.2 ng/L) [26]. In study coded S5 conducted by Kladar et al., focusing on measuring “total Δ9-THC, cannabidiol (CBD), and cannabinol (CBN) levels” in hemp food items, 17 cold-pressed hemp oils were examined. These products were primarily found in the markets of countries not evaluated by the European Food Safety Authority (EFSA). According to the content analysis of the oils, 14 out of 17 of these oils were reported to contain Δ9-THC above the LOQ (1 mg/kg). This study addressing cannabinoid exposure assessment states that the consumption of 75% of cannabis oil samples consumed by adults leads to a “total” Δ9-THC intake of more than 1 µg/kg per body weight (bw). It also states that consumption of the same percentage of the collected samples would lead to excessive intakes (> 0.4 µg/kg bw) in adolescents (14–18 years) and that only two products can be safely consumed by adolescents [44]. In study coded S4, conducted with 32 participants who consumed 30 g of hemp oil per day, a study was conducted on the possibility of testing positive for cannabinoids in urine samples of short-term (1 week) and long-term (12 weeks) consumers. In this study, the drug screening test results, performed with immunoassay, on all urine samples in the short-term study were below the screening cut-off level (25 ng/mL), whereas in the long-term study, three urine samples collected from two participants (out of a total of 480 samples) were above the LOQ level for confirmation analysis, but all of these samples were negative in the screening analysis. The research highlighted that consuming normal quantities of hemp seed products available in Korea, whether for short or long periods, did not result in positive tests for cannabinoids in urine samples [48].

Limited data exist on the skin absorption of THC when applying creams containing hemp oil for cosmetic purposes. In study coded S3 conducted by Paul et al. (2019), on 10 healthy participants without any substance use in the UK, hemp oil was applied to their heads for a 6-week periods, and CBD, THC, THC-OH, and THC-COOH were not detected in hair by GC-MS/MS after cosmetic application. However, THC was detected at trace amounts of 0.13, 0.04, 0.02 and 0.01 pg/mg respectively in 4 out of 10 participants [45]. In a study coded S1 designed on the possibility that the use of hemp oil in cosmetic products and various personal hygiene products may cause positive drug test results in humans, the results were aimed at explaining a real case of driving under the influence of drugs, which was claimed to be caused by the topical absorption of THC after the application of a cream containing hemp [49]. After three participants applied both creams to their skin every 2–4 h for 3 days, there were no positive findings regarding cannabinoids and metabolites in the urine and blood samples taken at certain time points. It has been emphasized that the frequent application of cosmetic products containing THC is unlikely to result in a positive drug test for cannabinoids in the blood or urine.

In this review, there are ***3 studies (S6-S8) that showed clearly positive results in the drug test after the use of CBD and hemp oils***. In a case study coded S7 from Italy by Chinello et al. (2017), cannabinoid poisoning was reported in a preschool child who consumed prescribed hemp seed oil twice a day for 3 weeks to strengthen the immune system. In addition to all biochemical tests, a toxicological drug screening test was performed in the urine of the patients suspected of poisoning. It was negative for abused drugs (e.g., amphetamine and metamphetamine), while it was positive for cannabinoids (> 50 ng/mL). After 19 h, the result of the second toxicological screening analysis was positive for cannabinoids. Following medical care and observation, the child was discharged approximately 24 h later [46]. In this case, the lower limit of the THC concentration for cannabis seed oil to be considered illegal was below 0.5%. Although the THC concentration of the oil used in this study was detected 0.06%, cannabinoid intoxication occurred after ingestion. In another case report coded S6 about older adults or the elderly; after consuming various hemp food products made with hemp oil, people were referred to the emergency room for reasons such as nausea, vomiting, and dizziness. Several hours after digestion, blood samples were obtained. Two out of the 12 study participants required admission to intensive care due to depression of the central nervous system. All drug screening tests were positive for cannabinoids [47]. Another study S8, involving 23 hemp products, three of which were hemp oil containing THC, CBD, and THCA, examined 22 participants who consumed 2 tablespoons (10–15 g) of the oil orally every day. Urine samples were collected before dosing and at 8, 16, and 32 h after dosing. Only after 8 h, trace levels of THCA: 0.7 ng/ml and THC-COOH (1 ng/ml) were detected in the samples [50]. S8 was designed on hemp oils as one of the products that may affect performance in terms of doping. Even if the analysis results are below the LOQ, the trace amounts obtained pose a risk due to the doping regulations. According to the World Anti-Doping Agency regulations, there is a zero-tolerance regulation for all natural and synthetic cannabinoids. Another remarkable result is the presence of prohibited cannabinoids (all natural and synthetic cannabinoids with the exception of cannabidiol) in 30% of the urine samples taken 8 h after hemp oil consumption, which may lead to an unintentional violation of anti-doping regulations [50, 51].

### Effect of pure CBD oral intake on THC test results in drug tests

**The four studies designed to evaluate the conversion of CBD to THC in human metabolism as a result of the use of CBD Oil products** are shown in Table 2. **Two out of four studies** emphasize that there will be no biotransformation or extraction-induced transformation after the use of CBD and hemp oils. In study S9, simulated gastric fluid experiments indicated that orally administered CBD undergoes substantial acidic conversion to Δ9-THC and Δ8-THC, with more than 98% transformation occurring within 120 min. This chemical conversion underscores the potential for inadvertent in vivo THC exposure, which, depending on the sensitivity and specificity of the analytical methods employed, may result in positive outcomes in forensic and clinical drug testing frameworks [52]. In a study coded S12 conducted in Germany, nine volunteers were administered water-soluble CBD liquid (400 mg, single dose) with the lecithin emulsifier. In this study, blood and urine samples were collected at certain time points after application. All serum samples were negative; however, the urine specimens were only negative for THC-COOH at 0, 2, and 48 h. They observed reduced levels of ∆9-THC-COOH in the urine, and the highest THC-COOH value ​​was 17.9 ng/ml (> LOQ:10 ng/mL) after 6 h of ingestion. In contrast, two participants were positive for THC-COOH for up to 24 h [53]. In study S11, the evaluation of different CBD formulations under simulated gastric conditions revealed that water-soluble CBD exhibited up to a 100-fold higher conversion rate to Δ9-THC compared to CBD oil, although the absolute levels remained below the typical cut-off observed in commercial “THC-free” products. Based on the estimated daily intake scenario, the magnitude of in vivo conversion appears insufficient to trigger positive results in standard urinary cannabinoid testing, yet the findings highlight formulation-dependent variability in conversion potential, which is relevant for forensic interpretation [54]. Crippa et al. (2020) conducted a study coded S10, that sought to demonstrate whether oral cannabidiol converts to ∆8-THC, an isomer of ∆9-THC, or ∆9-THC in humans, 120 participants were given a dose of 300 mg of CBD. Blood samples were collected under two dietary conditions: one group fasted and then ate a standard meal 30 min before CBD administration, while the other group did not receive a standard breakfast. No ∆8-THC or ∆9-THC was found in the whole blood 3 and 6 h after oral CBD administration [55].

## Discussion

This review evaluates the potential of CBD and hemp oil consumption to influence routine drug tests across different biological matrices. The critical factor is not the THC content of a product, but whether its total concentration-declared or not-is sufficient to produce a positive test in blood, urine, or hair. A majority of the studies analyzing biological specimens following CBD or hemp oil consumption (S1-S8) found no detectable cannabinoids above standard cut-offs. The few positive results (S6-S8) were predominantly from case reports of acute intoxication [46, 47]. For example, one report documented detectable THC (0.06%) in a pediatric patient from a product that was within the legal limit [46]. Another study (S6) reported that 12 elderly individuals who consumed cookies prepared with hemp oil all tested positive for cannabinoids in the urine screening. Confirmatory serum analysis detected cannabinoids in seven of these individuals, with toxicological evaluation ruling out other psychoactive substances or alcohol. These incidents demonstrate that even legally compliant products can cause unintentional exposure and positive tests [47].

In studies S1–S5, cannabinoid concentrations in biological specimens following CBD or hemp oil use were generally below the cut-off or LOQ. However, isolated reports of measurable THC or its metabolites levels indicate that atypical exposure scenarios or product mislabeling may lead to detectable outcomes. This is particularly critical in zero-tolerance settings, where even concentrations near decision limits such as workplace testing or anti-doping programs, can lead to sanctions, underscoring the need for careful interpretation of borderline results [26, 44, 45, 48, 49]. A pertinent example comes from study S1, where a blood sample from a driver during a traffic stop revealed measurable THC and metabolites. The individual attributed this to the frequent use of topical hemp-based creams. However, when these same creams were applied under controlled conditions in the study, subsequent blood and urine samples showed no cannabinoid concentrations above the limits of detection (Blood LOD_THC_: 0.40 ng/mL, LOD_11−OH−THC_: 0.28 ng/mL, LOD_THC−COOH_:1.6 ng/mL; Urine LOD: LOD_THC−COOH_:1.2 ng/mL). This discrepancy suggests that the initial positive result was more likely due to systemic exposure from another source, product mislabeling, or differences in dosage, rather than routine dermal application [49]. Consequently, the increasing use of hemp-derived products may give rise to a “CBD oil defense”, analogous to the “poppy seed defense [57]. While current evidence, including the findings from S1, does not support the claim that transdermal application leads to detectable levels, the very possibility of such defenses highlights the urgent need for careful result interpretation and standardized testing protocols to ensure accurate conclusions.

Across the included studies, the impact of CBD and hemp oil use on drug testing outcomes varied substantially by the biological matrix analyzed. In urine, most controlled administration studies (S1–S5) reported cannabinoid concentrations below the screening cut-offs or trace levels close to analytical limits. This suggests that the routine consumption of regulated hemp products is unlikely to yield positive results. For instance, study S2 demonstrated that hemp oil consumption did not produce positive urine screens; while trace THCA was sometimes detected, this cannabinoid is not targeted in standard workplace or roadside protocols and thus does not influence interpretation. However, trace THC-COOH findings near the decision levels indicate a potential for borderline or positive interpretations, particularly in zero-tolerance settings (e.g., anti-doping). In blood, standard oral intake resulted in low or non-quantifiable THC or metabolites levels, whereas clearly positive results were primarily linked to intoxication or product mislabeling. In oral fluid, cannabinoid detection was absent following CBD oil and hemp oil use, aligning with its shorter detection window. In contrast, hair testing demonstrated the occasional presence of minimal metabolites after prolonged exposure (S3), highlighting how environmental or cosmetic contamination could be misinterpreted as active use in forensic contexts. Overall, while regulated CBD and hemp oils are unlikely to yield positive results under typical use conditions, unregulated products, prolonged consumption, and matrices with high interpretive uncertainty present a measurable risk of false-positive, borderline, or ambiguous results. This underscores the necessity for confirmatory testing and rigorous product composition oversight.

Recent studies have debated the potential for CBD to be converted into THC under acidic conditions, a reaction with significant implications for drug testing [12, 58–62]. This review evaluated this possibility based on studies S9-S12. A foundational in vitro study (S9) demonstrated that CBD could be converted to THC in simulated gastric fluid (pH ~ 1.2) using surfactants such as sodium dodecyl sulfate (SDS) [52]. Other in vitro studies have replicated these findings, showing conversion under specific, strongly acidic conditions [8, 37, 38, 52, 53, 62]. However, the non-physiological nature of these conditions has been heavily contested. Nahler et al. (2017) argued that such findings are misleading, citing a lack of evidence for in vivo conversion and asserting that the reaction is confined to artificial environments [60]. Subsequent in vivo studies support this critique. In study 12, Franz et al. (2023) administered 400 mg of a water-soluble CBD formulation to nine participants and detected no THC or its metabolites in the blood; trace urinary findings were attributed to product contamination rather than conversion [53]. The authors concluded that poor CBD solubility in the gastric fluid prevents significant conversion without solubilizing agents. Similarly, in study S10, Crippa et al. (2020) found no Δ9-THC or Δ8-THC in plasma after giving 120 volunteers a 300 mg CBD oral solution [55]. Although animal studies were not included in this systematic review, several were cited by human studies to support their findings. For instance, Crippa et al. (2020) referenced work by Palazzoli et al. (2017), in which male rats administered 50 mg/kg of CBD oil showed no detectable THC or its metabolites in the blood [63]. Similarly, a study conducted by Wray et al.(2017) found no THC or 11-OH-THC in pigs given 100 mg/mL of synthetic CBD [64]. Collectively, these data reinforce the conclusion that significant in vivo conversion of CBD to THC is unlikely under typical physiological conditions.

In addition to the studies included in this systematic review, a keyword search identified 16 studies that focused on the labeling and content analysis of oil-based cannabis products. Although excluded from the primary analysis of review for not directly addressing drug test outcomes, these studies provide a critical context on global product integrity, a major factor influencing test results. The analyzed products (*n* = 726) originated from numerous countries, including Argentina (*n* = 360), the United States (*n* = 111), and across Europe (*n* = 158), with 67 samples lacking a declared country of origin [24, 34, 35, 43, 44, 65–69]. A comparison of the label claims and the analytical results revealed widespread inaccuracies. Among the oils, 124 provided no content specification, and only 11 of those with specifications were found to be labeled compliantly. Notably, THC was detected in 455 (62.7%) of the oils analyzed. Several studies highlighted extreme cases. Eunyoung et al. (2020) reported an unlabeled hemp oil in Korea containing 19.73 mg/mL (approximately 2%) of THC, the highest concentration observed [70], while Takashina et al. (2022, 2023) documented significant content discrepancies in CBD oils from the Japanese market [71, 72]. Most alarmingly, synthetic cannabinoids were detected in four oil-based products from the United States [68].

The lack of standardized global regulations complicates the interpretation of drug test results. The legal status of cannabis-derived products at the time of the included studies, summarized in Tables 1 and 2, demonstrates significant variations across countries. Regulations differ not only between nations but also between states or provinces within the same country. A key point of divergence is the legal definition of “industrial hemp”. Some jurisdictions set a maximum THC concentarion of 1% in the leaves and flowering heads [73], while others impose a stricter limit of 0.5% [74]. National regulatory frameworks also differ substantially. In Canada, for instance, products such as hemp seeds and oil are exempt from licensing if they contain less than 10 µg/g THC. Conversely, products derived from other plant parts require licenses for medicinal and scientific use and specific import-export permits [75]. This regulatory patchwork raises concerns about the validity of drug test results and poses a legal risk to consumers, both domestically and during international travels. The absence of clear regulations in many countries further exacerbates these challenges.

## Conclusion

This systematic review demonstrates that cannabinoid-positive drug test results following the use of compliant CBD and hemp oil products are rare. Positive findings are primarily associated with products containing higher-than-declared or illegal levels of THC. The likelihood of a positive result is also influenced by the biological matrix and analytical method; for instance, oral fluid is susceptible to direct contamination, while highly sensitive urine assays with low cut-off values can detect trace exposures. Blood and hair analyses also present a risk of detection, particularly following chronic or high-dose use.

To improve the accuracy of test interpretation and mitigate legal challenges, it is critical to select a matrix appropriate for the intended detection window and to employ confirmatory analyses with a low limit of quantification (LOQ). Future research should concurrently analyze CBD, its metabolites, and the full cannabinoid profile in biological specimens from product users. This comparative approach is essential for distinguishing between inadvertent exposure to low-THC products and the deliberate use of high-THC cannabis.
